# Supplementary material for: Detection of PatIent-Level distances from single cell genomics and pathomics data with Optimal Transport (PILOT)
Source: Mol Syst Biol. 2023 Dec 19;20(2):57–74. doi: 10.1038/s44320-023-00003-8 (PMC10883279; doi:10.1038/s44320-023-00003-8)
Supplement: Supplementary file 1 — Appendix [file 44320_2023_3_MOESM1_ESM.pdf]

Appendix

Detection of PatIent-Level distances from single cell genomics and pathomics data with Optimal Transport (PILOT)

Mehdi Joodaki[1,@], Mina Shaigan[1,@], Victor Parra[1], Roman D. Bülow[2], Christoph Kuppe[3], David L. Hölscher[2], Mingbo Cheng[1], James S. Nagai[1], Michaël Goedertier[1,2], Nassim Bouteldja[2], Vladimir Tesar[5], Jonathan Barratt[6,7], Ian S.D. Roberts[8], Rosanna Coppo[9], Rafael Kramann[3,4], Peter Boor[2], Ivan G. Costa[1,*]

[1]Institute for Computational Genomics, Joint Research Center for Computational Biomedicine, RWTH Aachen University Medical School, [2]Institute of Pathology, Laboratory of Nephropathology, RWTH Aachen University Medical School, [3]Institute of Experimental Medicine and Systems Biology, RWTH Aachen University, [4]Department of Internal Medicine, Nephrology and Transplantation, Erasmus Medical Center, [5]Department of Nephrology, 1st Faculty of Medicine and General University Hospital, Charles University, Prague, Czech Republic, [6]John Walls Renal Unit, University Hospital of Leicester National Health Service Trust, Leicester, United Kingdom, [7]Department of Cardiovascular Sciences, University of Leicester, Leicester, United Kingdom, [8]Department of Cellular Pathology, Oxford University Hospitals National Health Services Foundation Trust, Oxford, United Kingdom, [9]Fondazione Ricerca Molinette. Regina Margherita Children's University Hospital, Torino, Italy, [*]pboor@ukaachen.de, ivan.costa@rwth-aachen.de, [@]Shared first authorship

Table of Contents

Appendix Table S1 2
Appendix Table S2 2
Appendix Table S3 3
Appendix Table S4 4
Appendix Figure S1 5
Appendix Figure S2 6
Appendix Figure S3 7
Appendix Figure S4 8
Appendix Figure S5 9
Appendix Figure S6 10
Appendix Figure S7 11
Appendix Figure S8 12
Appendix Figure S9 13
Appendix Figure S10 13
Appendix Figure S11 14
Appendix Group information 14

Appendix Table S1. Benchmarking of clustering results (ARI and Silhouette Scores), and area under the PR curve (AUCPR). Results in bold indicate the best value per method.

**Methods**	**Pseudobulk**			**Phemd**			**Proportions**			**PILOT**			**Pro.Phate**
**Datasets/Metrics**	ARI	Sil	AUCPR	ARI	Sil	AUCPR	ARI	Sil	AUCPR	ARI	Sil	AUCPR	AUCPR
**PDAC**	**1.00**	**0.73**	**1.00**	0.51	0.04	0.87	0.51	0.56	**1.00**	**1.00**	0.59	**1.00**	**1.00**
**Lupus PBMC**	0.57	0.34	**0.92**	0.51	0.02	0.58	**0.70**	**0.39**	0.91	0.61	0.28	0.89	0.91
**Kidney IgAN (G)**	0.50	0.01	0.51	0.50	-0.04	0.34	0.51	0.00	0.55	**0.55**	**0.07**	**0.60**	0.27
**Myoc. Infarc**	**1.00**	**0.86**	**1.00**	0.47	-0.06	0.26	0.81	0.70	0.98	0.90	0.73	0.98	0.94
**Kidney AC (T)**	0.57	0.21	0.54	0.51	0.00	0.76	0.53	0.25	0.87	**0.68**	**0.30**	**0.93**	0.88
**Kidney AC (G)**	0.51	0.25	0.59	0.51	-0.01	0.66	0.57	0.25	**0.96**	**0.64**	**0.36**	**0.96**	**0.96**
**COVID-19**	0.51	-0.25	0.92	0.51	**-0.06**	0.88	0.52	-0.14	**0.95**	**0.55**	-0.14	0.94	0.93
**Kidney**	0.71	0.16	0.77	**0.76**	**0.44**	**1.00**	0.70	0.24	0.92	0.62	0.11	0.81	0.71
**Kidney IgAN (T)**	0.48	0.07	0.54	0.49	-0.03	0.48	0.56	0.00	0.54	**0.58**	**0.10**	**0.59**	0.56
**Lung**	**0.69**	-0.17	0.59	0.29	-0.02	0.85	0.67	-0.03	**0.90**	0.67	**-0.01**	**0.90**	0.57
**Diabetes**	**0.67**	**0.19**	**0.74**	0.62	-0.13	0.51	0.64	-0.03	0.50	**0.67**	-0.01	**0.71**	0.54
**Foll. lym.**	0.50	0.29	0.90	0.52	0.09	0.97	0.48	**0.95**	**1.00**	**0.76**	0.60	**1.00**	**1.00**

Appendix Table S2. Benchmarking of Ordered Trajectory Analysis (Spearman Correlation). Spearman correlation values in bold indicate the best performing method per data set.

**Datasets/Metrics**	**Pseudobulk**	**Phemd**	**Pro. Phate**	**Proportions**	**PILOT**
**Myoc. Infarc**	**0.83**	-0.15	0.77	0.81	0.81
**PDAC**	**0.80**	0.34	**0.80**	**0.80**	**0.80**
**Lupus PBMC**	0.62	0.11	0.61	**0.66**	0.55
**Diabetes**	0.34	-0.30	-0.20	0.45	**0.55**
**Foll. lym.**	0.23	0.37	**0.58**	**0.58**	**0.58**
**Kidney**	0.50	**0.91**	0.22	0.73	0.66
**Lung**	-0.31	0.54	-0.28	0.52	**0.57**
**COVID-19**	0.16	-0.09	0.13	0.25	**0.26**
**Kidney AC (G)**	0.49	0.12	0.63	**0.64**	0.62
**Kidney AC (T)**	**0.57**	0.12	0.48	0.47	**0.57**
**Kidney IgAN (G)**	0.12	-0.09	0.31	0.37	**0.38**
**Kidney IgAN (T)**	0.26	0.27	**0.39**	0.36	**0.39**

Appendix Table S3. We used distinct statistical tests (Chi-Square for discrete and ANOVA for continuous variables) to evaluate association between detected clusters, biological or technical variables for all data sets. We only shown results with adjusted p-value < 0.05. The variables were sorted first by data and then by increasing p-values.

Data set	Variable	Test	P-value
**Myocardial Infarction**	Status	Chi-Squared	**3.990000e-4**
**Follicular Lymphoma**	Status	Chi-Squared	**2.057900e-2**
**Diabetes**	Status	Chi-Squared	**1.480000e-4**
**Diabetes**	Strain	Chi-Squared	**3.190000e-4**
**Diabetes**	Development stage	Chi-Squared	**1.481000e-3**
**PDAC**	Status	Chi-Squared	**2.510999e-8**
**PDAC**	Staging	Chi-Squared	**4.156157e-5**
**PDAC**	pathologic_diagnoses	Chi-Squared	**4.719115e-3**
**PDAC**	Location	Chi-Squared	**3.329660e-2**
**PDAC**	Diabetes	Chi-Squared	**3.995506e-2**
**PDAC**	vascular_invasion	Chi-Squared	**4.639490e-2**
**Lupus**	Status	Chi-Squared	**6.020011e-15**
**Lupus**	Processing_Cohort	Chi-Squared	**4.852031e-6**
**Lupus**	batch_cov	Chi-Squared	**3.462091e-4**
**Kidney**	Status	Chi-Squared	**1.590000e-4**
**Kidney**	diabetes_history	Chi-Squared	**9.195000e-3**
**Kidney**	BMI	Chi-Squared	**4.905700e-2**
**Lung**	study	Chi-Squared	**3.067563e-25**
**Lung**	Status	Chi-Squared	**9.319169e-13**
**Lung**	origin	Chi-Squared	**1.630440e-12**
**Lung**	ever_smoker	Chi-Squared	**1.765099e-2**
**Lung**	sex	Chi-Squared	**2.977980e-2**
**Kidney IgAN Tubuli**	Status	Chi-Squared	**1.450070e-17**
**Kidney IgAN Tubuli**	Sex	Chi-Squared	**1.407880e-2**
**Kidney IgAN Tubuli**	Age	ANOVA	**9.756758e-8**
**Covid**	City	Chi-Squared	**2.300698e-9**
**Covid**	Status	Chi-Squared	**6.018506e-4**
**Covid**	Age	ANOVA	**4.270000e-4**
**Covid**	Sampling day (Days after symptom onset)	ANOVA	**1.863300e-2**
**Kidney IgAN Glomeruli**	Status	Chi-Squared	**1.057934e-15**
**Kidney IgAN Glomeruli**	Age	ANOVA	**1.682000e-3**

Appendix Table S4. Log likelihood test and hazard ratios (HR) of cox regression for disease progression and morphometric variables

Features	p_value	HR	HR_lower_95	HR_higher_95
Disease_Progression	2.38E-11	14.22285964	6.133761376	32.97972059
Tuft-Area-Fraction	6.55E-06	0.006096	0.000754147	0.049275825
Age	0.000161201	1.026118273	1.012515212	1.03990409
Tuft Elongation	0.035174436	0.995855111	0.991024645	1.000709122
Tuft Area	0.059838632	0.99999991	0.999999796	1.000000024
Glomerular Area	0.084688986	0.99999995	0.999999883	1.000000017
Tuft Circularity	0.120647357	0.997847514	0.99474223	1.000962492
Tuft Eccentricity	0.193320223	0.99911608	0.99765839	1.0005759
Bowman Area	0.285387443	0.999999915	0.999999741	1.000000088
Glomerular Circularity	0.292065233	0.998989099	0.996929139	1.001053316
Glomerular Diameter	0.295112885	0.999996112	0.999988267	1.000003957
Tubular Distance	0.334379046	0.999806135	0.999357973	1.000254498
Tubular Area	0.361417305	1.000000128	0.999999865	1.000000392
Tuft Solidity	0.468203891	0.999443691	0.997818499	1.001071531
Glomerular Distance	0.55738282	1.000000526	0.999998905	1.000002148
Glomerular Solidity	0.594992091	0.999714882	0.998620471	1.000810492
Glomerular Eccentricity	0.721397871	1.000225975	0.999008779	1.001444655
Glomerular Elongation	0.944083375	0.999862207	0.995995708	1.003743715
Tubular Diameter	0.954027614	0.999999227	0.999972858	1.000025597

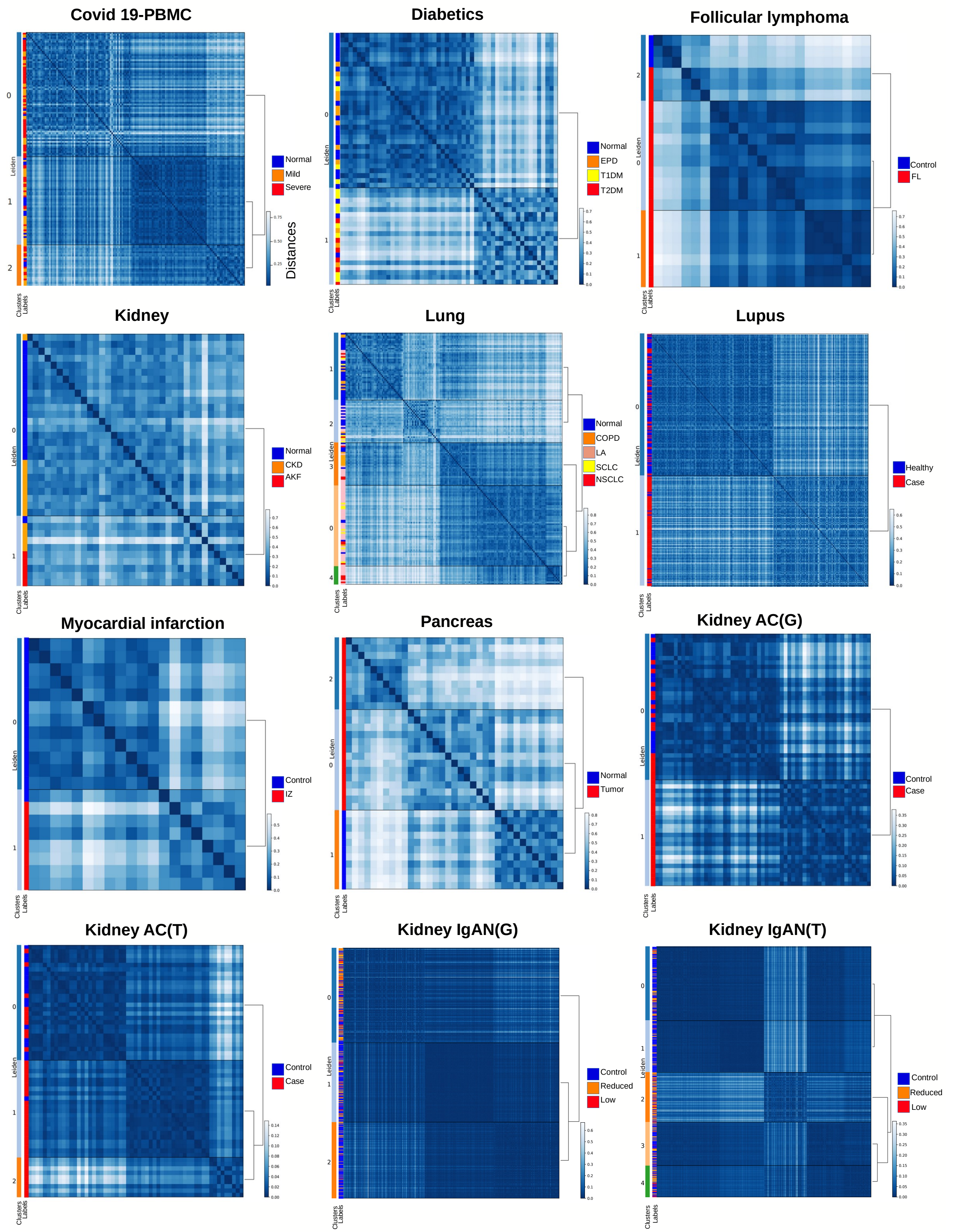

Appendix Figure S1. Heatmap representation of PILOT estimated Wasserstein distance and clustering using the Leiden algorithm. Color bars in the left of each heatmap shown respectivelly the cluster and the class labels.

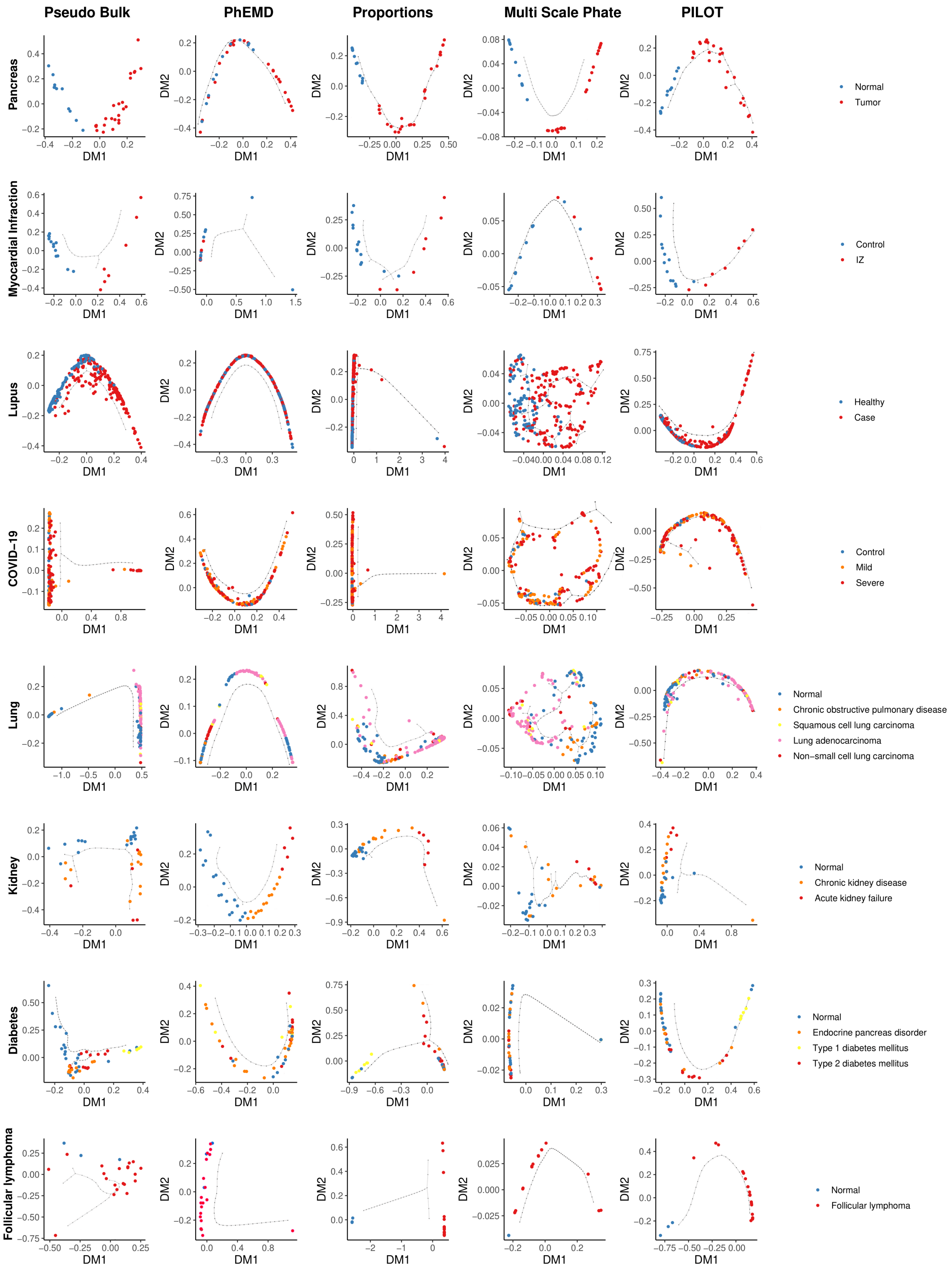

Appendix Figure S2. Diffusion maps of single cell data sets (rows) for Pseudo Bulk, PhEMD, Proportions, Multiscale Phate, and PILOT (columns). Doted lines represent backbones of the estimated trajectories.

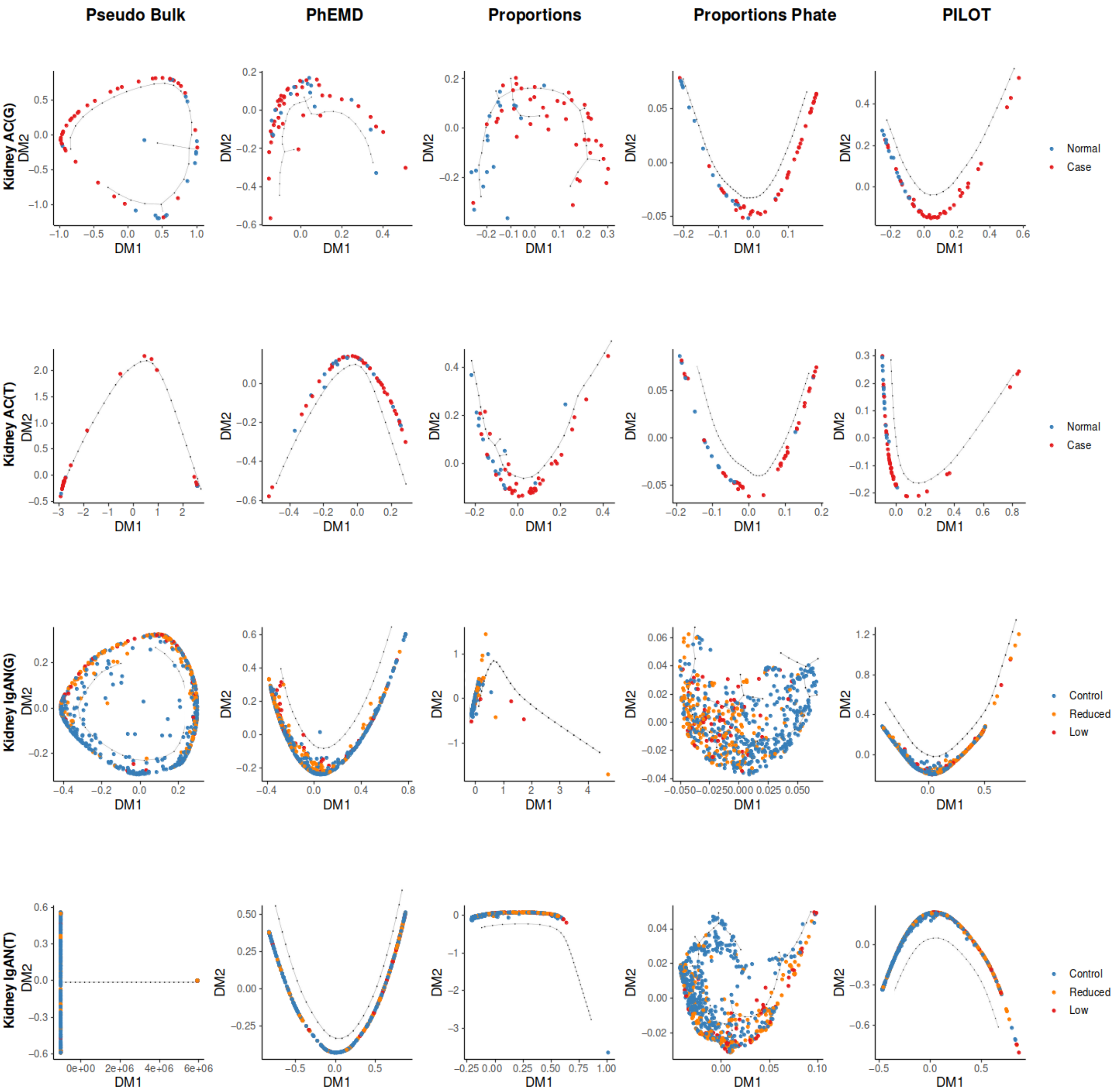

Appendix Figure S3. Diffusion maps of pathomics data sets (rows) for Pseudo Bulk, PhEMD, Proportions, Multiscale Phate, and PILOT (columns). Doted lines represent backbones of the estimated trajectories.

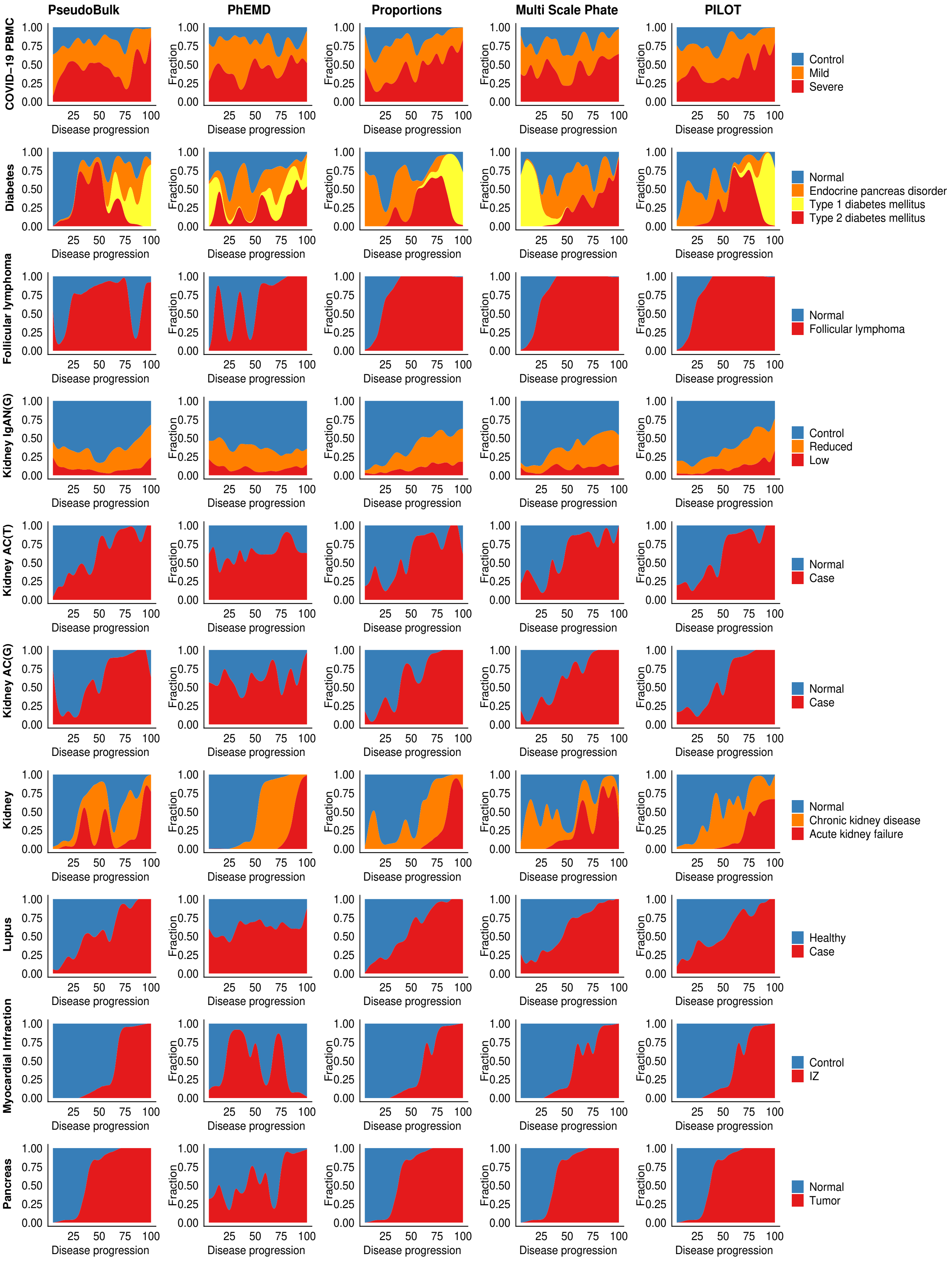

Appendix Figure S4. Fraction samples (y-axis) for distinct sample labels over the pseudo-time (x-axis) for disease progression trajectories.

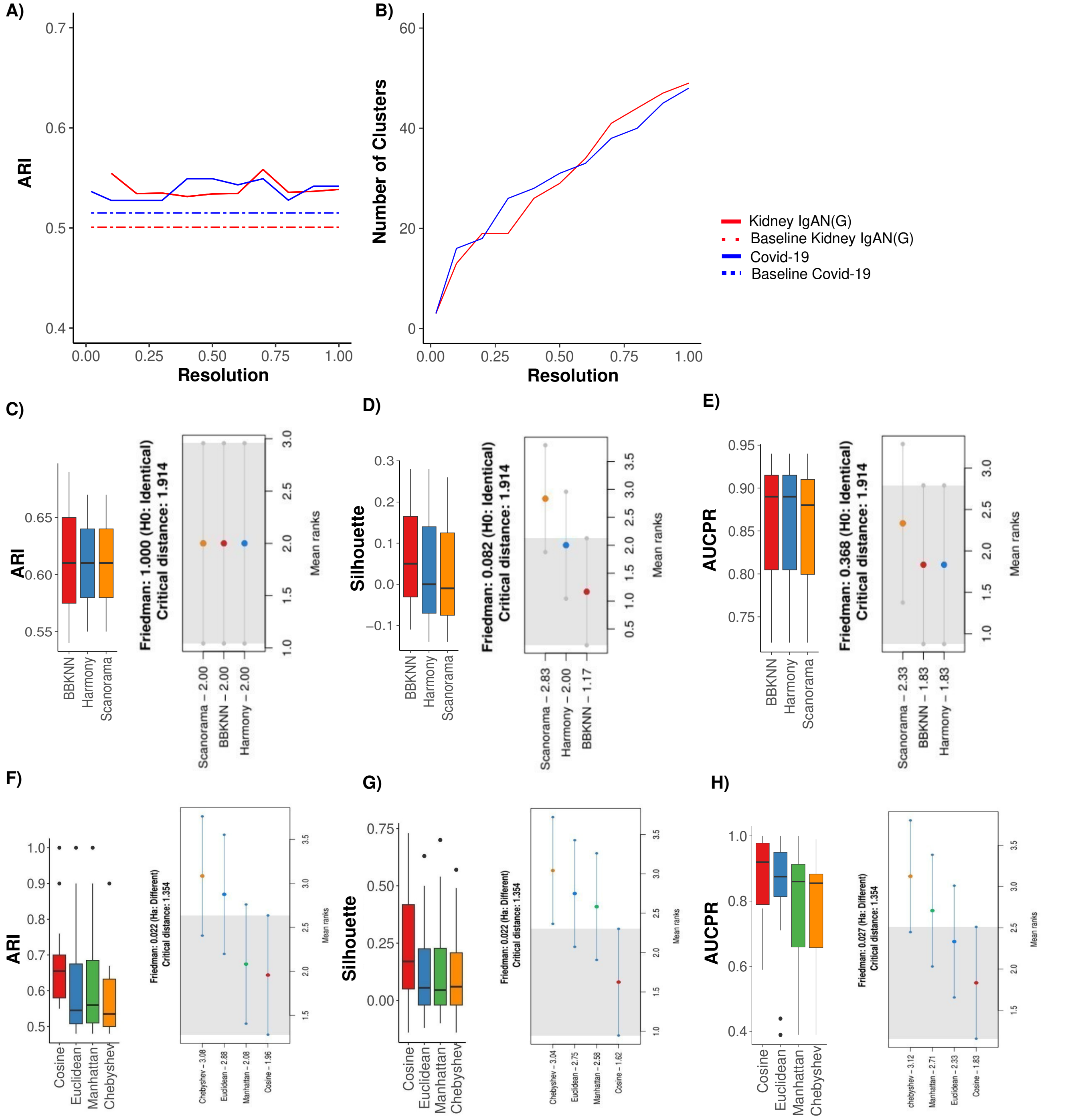

Appendix Figure S5. A - Clustering performance (measured by ARI) for PILOT (solid lines) and pseudo-bulk (traced lines) for distinct cluster resolution. We observe that for both Kidney IgAN and COVID-19, the number of clusters did not impacted the results from PILOT. B - Number of clusters for distinct Leiden algorithm resolution parameters. C-E - Benchmarking on the effect of distinct batch correction methods for data sets, where raw count matrices were available (Lupus, COVID, and Diabetes). F-H Benchmarking on the use of distinct distance/similarity metrics as cost matrix C for EMD. Low ranking values in the Friedman scores indicates best performing approaches.

A) Healthy cardiomyocytes

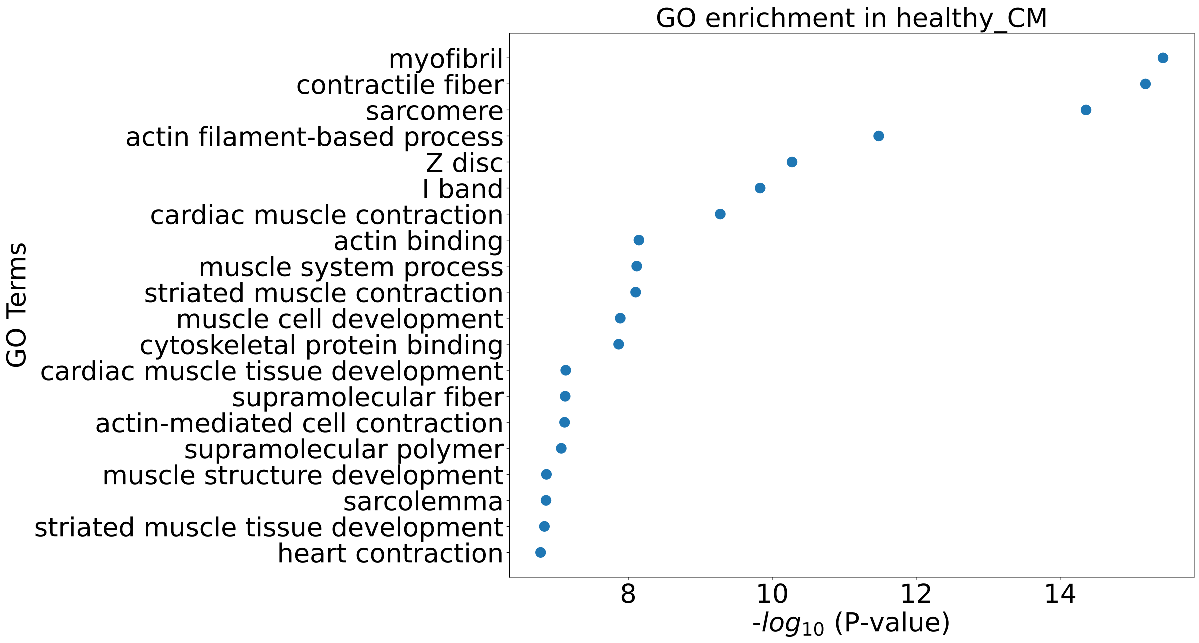

B) Myofibroblasts

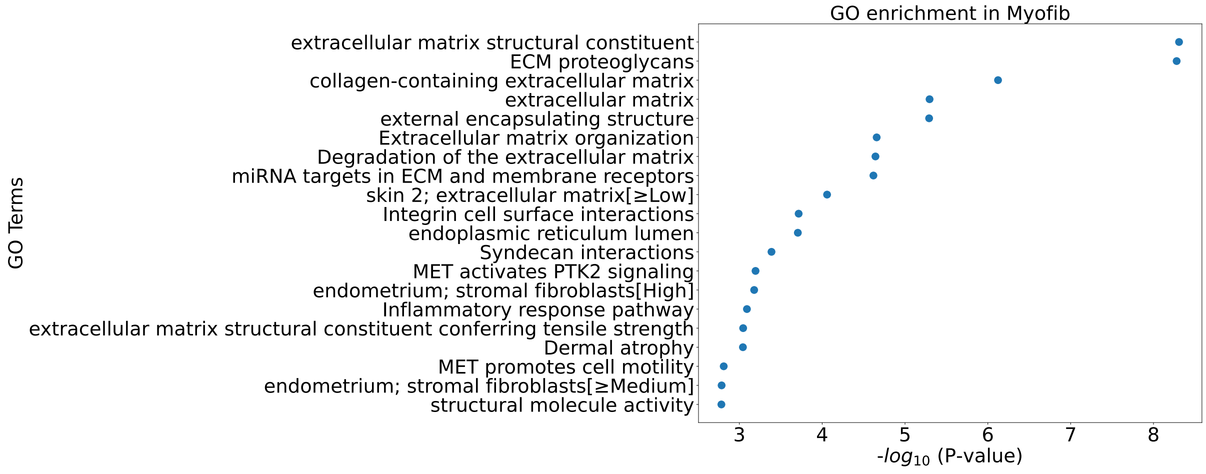

Appendix Figure S6. Top 20 terms of the enrichment analysis by using selected genes for Healthy cardiomyocytes and myofibroblasts (based on the first 50 genes with FC > 0.5 and Wald-test p-value < 0.01). The analysis was performed with g:profiler https://biit.cs.ut.ee/gprofiler/gost.

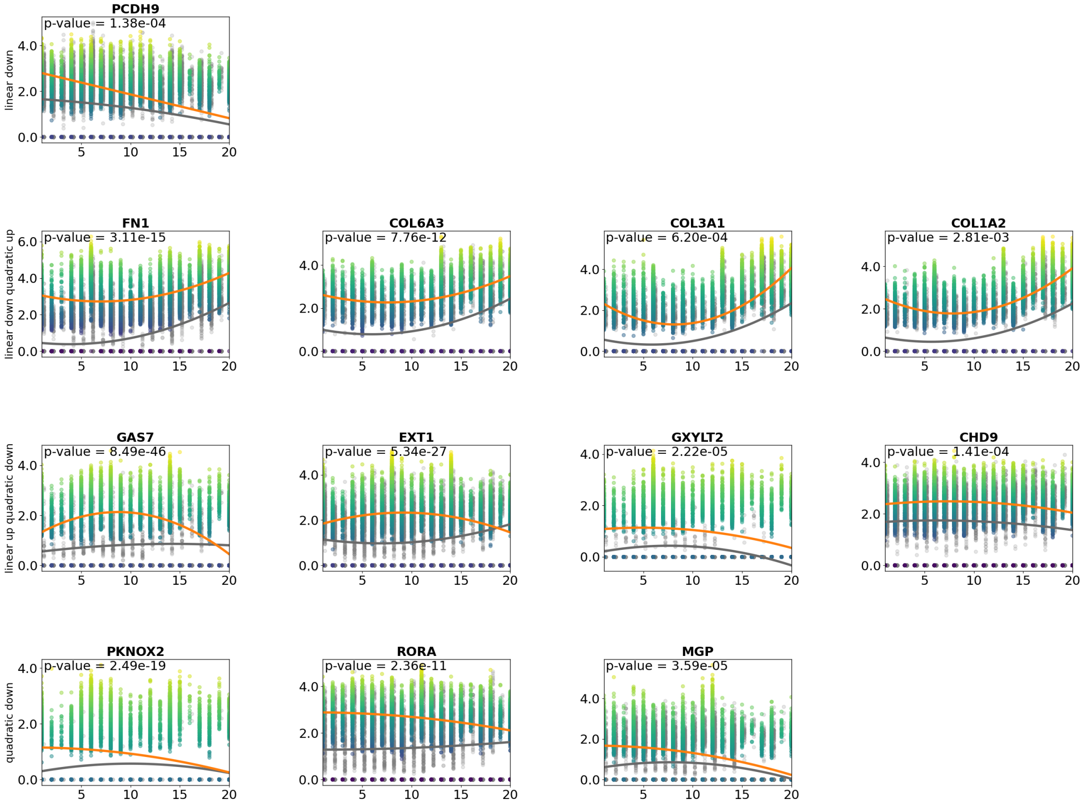

Appendix Figure S7. Top genes associated (FC > 0.5 and ranked by p-value of the Wald-test) from Myofibroblasts of the Myocardial infarction scRNA-seq. Every line corresponds to distinct models (linear, quadratic, linear-quadratic) and patterns (up vs. down). Only significant genes (p-value < 0.01) are shown.

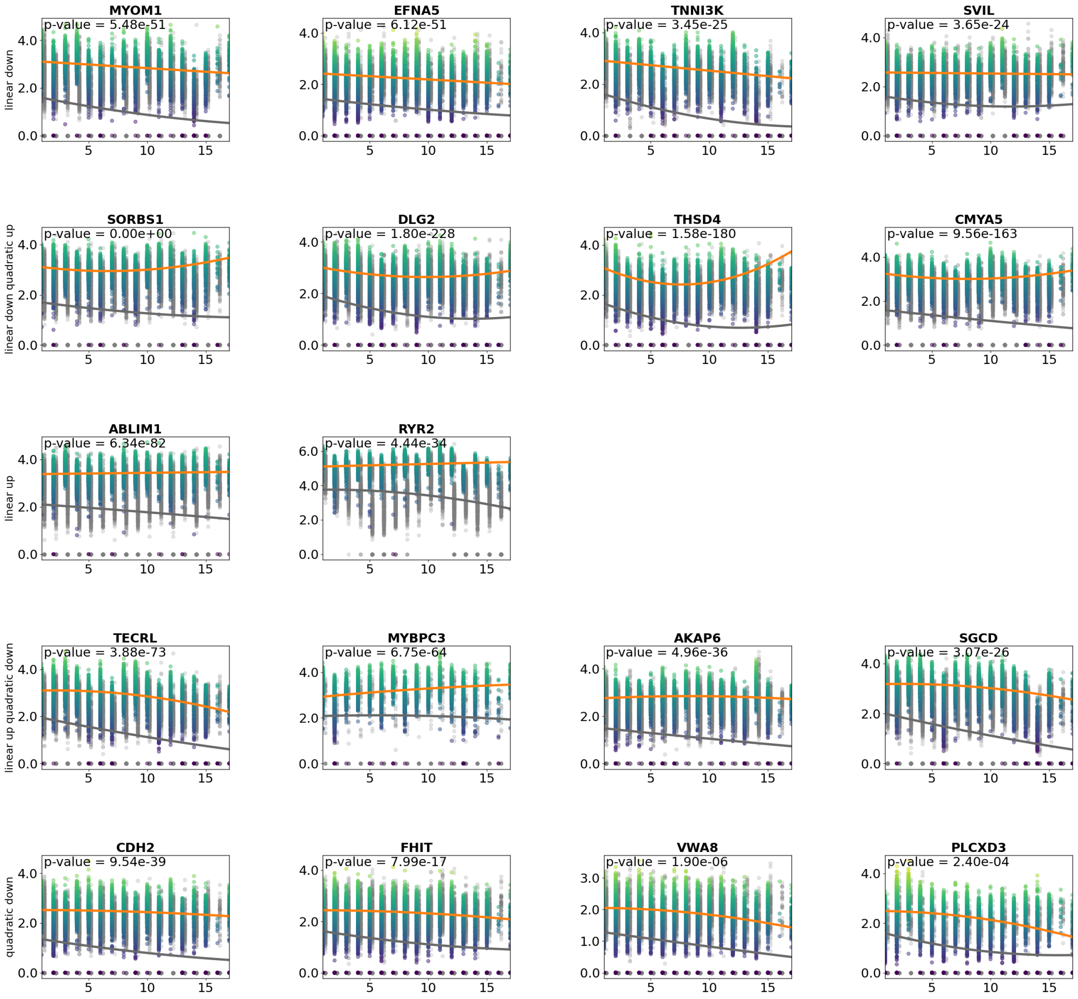

Appendix Figure S8. Top genes associated (FC > 0.5 and ranked by p-value of the Wald-test) from Healthy cardiomiocytes of the myocardial infarction scRNA-seq. Every line corresponds to distinct models (linear, quadratic, linear-quadratic) and patterns (up vs. down). Only significant genes (p-value < 0.01) are shown.

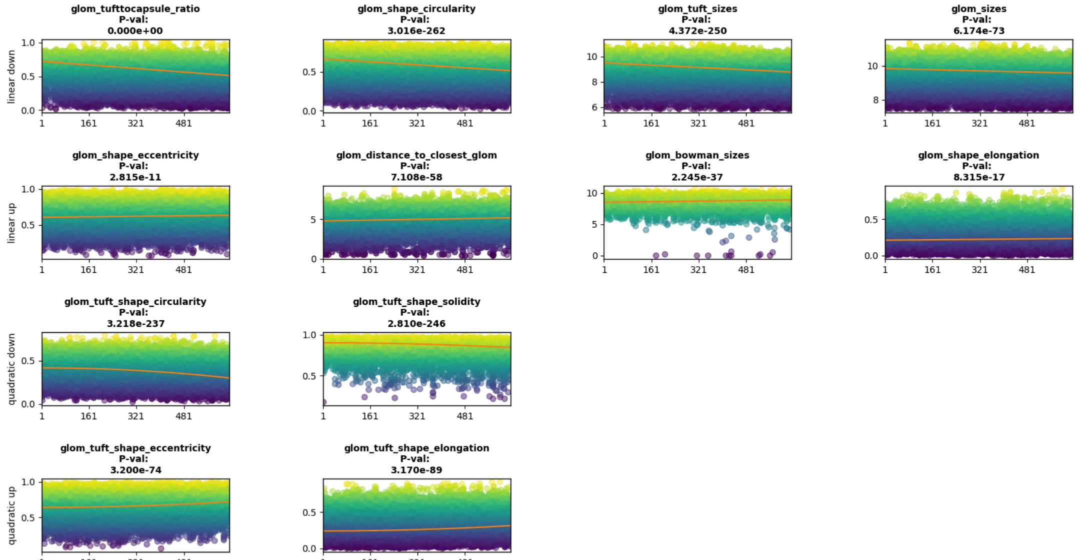

Appendix Figure S9. Top morphological features associated (ranked by adjusted R2) from Kidney IgA gromeruli. Every line corresponds to distinct models (linear, quadratic, linear-quadratic) and patterns (up vs. down). Significant features (p-value < 0.05) are marked in bold.

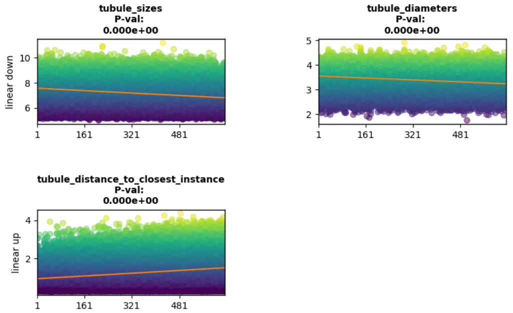

Appendix Figure S10. Top morphological features associated (ranked by adjusted R2) from Kidney IgA tubule. Every line corresponds to distinct models (linear, quadratic, linear-quadratic) and patterns (up vs. down). Significant features (p-value $<$ 0.05) are marked in bold.

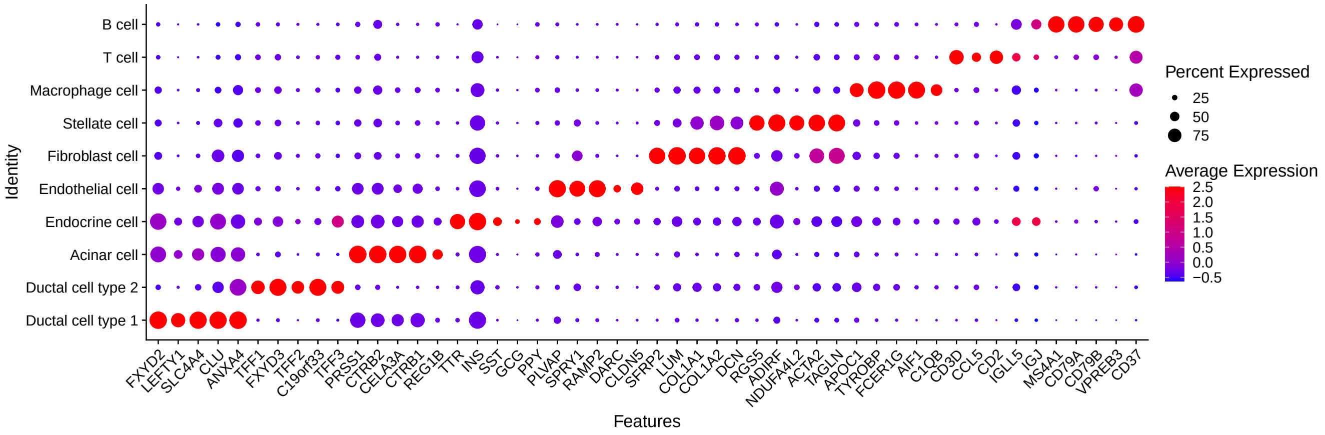

Appendix Figure S11. Markers used to characterize clusters in the PDAC single cell data set.

Appendix Group information

Validation Study of the Oxford Classification of IgAN (VALIGA) investigators:

M.L. Russo (MA, PhD, Fondazione Ricerca Molinette, Torino, Italy); S. Troyanov (MD, Division of Nephrology, Department of Medicine, Hopital du Sacre-Coeur de Montreal, Montreal, Quebec, Canada); H.T. Cook (MD, Centre for Complement and Inflammation Research, Department of Medicine, Imperial College, London, England); I. Roberts (MD, Department of Cellular Pathology, Oxford University Hospitals NHS Foundation Trust, John Radcliffe Hospital, Oxford, United Kingdom); V. Tesar, (MD, Department of Nephrology, 1st Faculty of Medicine and General University Hospital, Charles University, Prague, Czech Republic); D. Maixnerova (MD, Department of Nephrology, 1st Faculty of Medicine and General University Hospital, Charles University, Prague, Czech Republic); S. Lundberg (MD, Nephrology Unit, Department of Clinical Sciences, Karolinska Institute, Stockholm, Sweden); L. Gesualdo (MD, Department of Nephrology, Emergency and Organ Transplantation, University of Bari "Aldo Moro," Foggia-Bari, Italy); F. Emma (MD, Division of Nephrology, Department of Pediatric Subspecialties, Bambino Gesù Children's Hospital IRCCS, Rome, Italy); F. Diomedi (MD, Division of Nephrology, Department of Pediatric Subspecialties, Bambino Gesù Children's Hospital IRCCS, Rome, Italy); G. Beltrame (MD, Nephrology and Dialysis Unit, San Giovanni Bosco Hospital, and University of Turin,Turin, Italy); C. Rollino (MD, Nephrology and Dialysis Unit, San Giovanni Bosco Hospital, and University of Turin,Turin, Italy); A. Amore (MD, Nephrology Unit, Regina Margherita Children's Hospital,Turin, Italy); R. Camilla (MD Nephrology Unit, Regina Margherita Children's Hospital, Turin, Italy); L. Peruzzi (MD, Nephrology Unit, Regina Margherita Children's Hospital, Turin, Italy); M. Praga (MD, Nephrology Unit, Hospital 12 de Octubre,Madrid, Spain); S. Feriozzi (MD, Nephrology Unit, Belcolle Hospital, Viterbo, Italy), R. Polci, (MD, Nephrology Unit, Belcolle Hospital,Viterbo, Italy); G. Segoloni, (MD, Division of Nephrology Dialysis and Transplantation, Department of Medical Sciences, Città della Salute e della Scienza Hospital and University of Turin, Turin, Italy); L.Colla (MD, Division of Nephrology Dialysis and Transplantation, Department of Medical Sciences, Città della Salute e della Scienza Hospital and University of Turin,Turin, Italy); A. Pani (MD, Nephrology Unit, G. Brotzu Hospital, Cagliari, Italy); D. Piras (MD, Nephrology Unit, G. Brotzu Hospital, Cagliari, Italy), A. Angioi (MD, Nephrology Unit, G. Brotzu Hospital, Cagliari, Italy); G. Cancarini, (MD, Nephrology Unit, Spedali Civili University Hospital, Brescia, Italy); S. Ravera (MD, Nephrology Unit, Spedali Civili University Hospital, Brescia, Italy); M. Durlik (MD, Department of Transplantation Medicine, Nephrology, and Internal Medicine, Medical University ofWarsaw,Warsaw, Poland); E. Moggia (Nephrology Unit, Santa Croce Hospital, Cuneo, Italy); J. Ballarin (MD, Department of Nephrology, Fundacion Puigvert, Barcelona, Spain); S. Di Giulio (MD, Nephrology Unit, San Camillo Forlanini Hospital, Rome, Italy); F. Pugliese (MD, Department of Nephrology, Policlinico Umberto I University Hospital, Rome, Italy); I. Serriello (MD, Department of Nephrology, Policlinico Umberto I University Hospital, Rome, Italy); Y. Caliskan (MD, Division of Nephrology, Department of Internal Medicine, Istanbul Faculty of Medicine, Istanbul University, Istanbul, Turkey); M. Sever (MD, Division of Nephrology, Department of Internal Medicine, Istanbul Faculty of Medicine, Istanbul University, Istanbul, Turkey); I. Kilicaslan (MD, Department of Pathology, Istanbul Faculty of Medicine, Istanbul University, Istanbul, Turkey); F. Locatelli (MD, Department of Nephrology and Dialysis, Alessandro Manzoni Hospital, ASST Lecco, Italy); L. Del Vecchio (MD, Department of Nephrology and Dialysis, Alessandro Manzoni Hospital, ASST Lecco, Italy); J.F.M.Wetzels (MD, Departments of Nephrology, Radboud University Medical Center, Nijmegen, the Netherlands); H. Peters (MD, Departments of Nephrology, Radboud University Medical Center, Nijmegen, the Netherlands); U. Berg (MD, Division of Pediatrics, Department of Clinical Science, Intervention and Technology, Huddinge, Sweden); F. Carvalho (MD, Nephrology Unit, Hospital de Curry Cabral, Lisbon, Portugal); A.C. da Costa Ferreira (MD, Nephrology Unit,

Hospital de Curry Cabral, Lisbon, Portugal); M. Maggio (MD, Nephrology Unit, Hospital Maggiore di Lodi, Lodi, Italy); A. Wiecek (MD, Department Nephrology, Endocrinology and Metabolic Diseases, Silesian University of Medicine, Katowice, Poland); M. Ots-Rosenberg(MD, Nephrology Unit, Tartu University Clinics, Tartu,Estonia); R. Magistroni (MD, Department of Nephrology, Policlinic of Modena and Reggio Emilia; Modena, Italy); R. Topaloglu (MD, Department of Pediatric Nephrology and Rheumatology, Hacettepe University, Ankara, Turkey); Y. Bilginer (MD, Department of Pediatric Nephrology and Rheumatology, Hacettepe University, Ankara, Turkey); M. D'Amico (MD, Nephrology Unit, S. Anna Hospital, Como, Italy); M. Stangou (MD, Department of Nephrology, Hippokration General Hospital, Aristotle University of Thessaloniki, Thessaloniki, Greece); F. Giacchino (MD, Nephrology Unit, Ivrea Hospital, Ivrea, Italy); D. Goumenos (MD Department of Nephrology, University Hospital of Patras, Patras, Greece); P. Kalliakmani (MD Department of Nephrology, University Hospital of Patras, Patras, Greece); M. Papasotiriou (MD Department of Nephrology, University Hospital of Patras, Patras, Greece); K. Galesic (MD, Department of Nephrology, University Hospital Dubrava, Zagreb, Croatia); C. Geddes (MD, Renal Unit,Western Infirmary Glasgow, Glasgow, United Kingdom); K. Siamopoulos (MD, Nephrology Unit, Medical School University of Ioanina, Ioannina, Greece); O. Balafa (MD, Nephrology Unit,Medical School University of Ioanina, Ioannina, Greece); M. Galliani (MD, Nephrology Unit, S.Pertini Hospital, Rome, Italy); P. Stratta (MD, Department of Nephrology, Maggiore della Carità Hospital, Piemonte Orientale University, Novara, Italy); M. Quaglia (MD, Department of Nephrology, Maggiore della Carità Hospital, Piemonte Orientale University, Novara, Italy); R. Bergia (MD, Nephrology Unit, Degli Infermi Hospital, Biella,Italy); R. Cravero (MD, Nephrology Unit, Degli Infermi Hospital, Biella, Italy); M. Salvadori, (MD, Department of Nephrology, Careggi Hospital, Florence, Italy); L. Cirami (MD, Department of Nephrology, Careggi Hospital, Florence, Italy); B. Fellstrom (MD, Renal Department, University of Uppsala, Uppsala, Sweden); H. Kloster Smerud (MD, Renal Department, University of Uppsala, Uppsala, Sweden); F. Ferrario (MD, Nephropathology Unit, San Gerardo Hospital, Monza, Italy); T. Stellato (MD, Nephropathology Unit, San Gerardo Hospital, Monza, Italy); J. Egido (MD, Department of Nephrology, Fundacion Jimenez Diaz, Madrid, Spain); C. Martin (MD, Department of Nephrology, Fundacion Jimenez Diaz, Madrid, Spain); J. Floege (MD, Nephrology and Immunology, Medizinische Klinik II, University of Aachen, Aachen, Germany); F. Eitner (MD, Nephrology and Immunology, Medizinische Klinik II, University of Aachen, Aachen, Germany); A. Lupo (MD, Department of Nephrology, University of Verona, Verona, Italy); P. Bernich (MD, Department of Nephrology, University of Verona, Verona, Italy); P. Menè (Department of Nephrology,S. Andrea Hospital, Rome, Italy); M. Morosetti (Nephrology Unit, Grassi Hospital, Ostia, Italy); C. van Kooten, (MD, Department of Nephrology, Leiden University Medical Centre, Leiden, The Netherlands); T. Rabelink (MD, Department of Nephrology, Leiden University Medical Centre, Leiden, The Netherlands); M.E.J. Reinders (MD, Department of Nephrology, Leiden University Medical Centre, Leiden, The Netherlands); J.M. Boria Grinyo (Department of Nephrology, Hospital Bellvitge, Barcelona, Spain); S. Cusinato (MD, Nephrology Unit, Borgomanero Hospital, Borgomanero, Italy); L. Benozzi (MD, Nephrology Unit, Borgomanero Hospital, Borgomanero, Italy); S. Savoldi, (MD, Nephrology Unit, Civile Hospital, Ciriè, Italy); C. Licata (MD, Nephrology Unit, Civile Hospital, Ciriè, Italy); M. Mizerska-Wasiak (MD, Department of Pediatrics, Medical University ofWarsaw,Warsaw, Poland); G. Martina (MD, Nephrology Unit, Chivasso Hospital, Chivasso, Italy); A. Messuerotti (MD, Nephrology Unit, Chivasso Hospital, Chivasso, Italy); A. Dal Canton (MD, Nephrology Unit, S. Matteo Hospital, Pavia, Italy); C. Esposito (MD, Nephrology Unit, Maugeri Foundation, Pavia, Italy); C. Migotto (MD, Nephrology Unit, Maugeri Foundation, Pavia, Italy); G. Triolo MD, Nephrology Unit CTO, Turin, Italy); F.Mariano (MD, Nephrology Unit CTO, Turin, Italy); C. Pozzi (MD, Nephrology Unit, Bassini Hospital, Cinisello Balsamo, Italy); R. Boero (MD, Nephrology Unit, Martini Hospital, Turin, Italy);

VALIGA pathology investigators: S. Bellur (MD, Department of Cellular Pathology, Oxford University Hospitals NHS Foundation Trust, John Radcliffe Hospital, Oxford, United Kingdom); G.Mazzucco (MD, Pathology Department, University of Turin, Turin, Italy); C. Giannakakis (MD, Pathology Department, La Sapienza University, Rome, Italy); E. Honsova (MD, Department of Clinical and Transplant Pathology, Institute for Clinical and Experimental Medicine, Prague, Czech Republic); B. Sundelin (MD Department of Pathology and Cytology, Karolinska University Hospital, Karolinska Institute, Stockholm, Sweden); A.M. Di Palma (Nephrology Unit, Aldo Moro University, Foggia-Bari, Italy); F. Ferrario (MD, Nephropathology Unit, San Gerardo Hospital, Monza, Italy); E. Gutiérrez (MD, Renal, Vascular and Diabetes Research Laboratory, Fundación Instituto de Investigaciones Sanitarias-Fundación Jiménez Díaz, Universidad Autónoma de Madrid, Madrid, Spain); A.M. Asunis (MD, Department of Pathology, Brotzu Hospital, Cagliari, Italy); J. Barratt (MD, The JohnWalls Renal Unit, Leicester General Hospital, Leicester, United Kingdom); R. Tardanico (MD, Department of Pathology, Spedali Civili Hospital, University of Brescia, Brescia, Italy); A. Perkowska-Ptasinska (MD, Department of Transplantation Medicine, Nephrology and Internal Medicine, Medical University of Warsaw, Warsaw, Poland); J. Arce Terroba (MD, Pathology Department, Fundació Puigvert, Barcelona, Spain); M. Fortunato (MD, Pathology Department, S. Croce Hospital, Cuneo, Italy); A. Pantzaki (MD, Department of Pathology, Hippokration Hospital, Thessaloniki, Greece); Y. Ozluk (MD, Department of Pathology, Istanbul University, Istanbul Faculty of Medicine, Istanbul, Turkey); E. Steenbergen (MD, Radboud University Medical Center, Department of Pathology, Nijmegen, The Netherlands); M. Soderberg (MD, Department of Pathology, Drug Safety and Metabolism, Huddinge, Sweden); Z. Riispere (MD, Department of Pathology, University of Tartu, Tartu, Estonia); L. Furci (MD, Pathology

Department, University of Modena, Italy); D. Orhan (MD, Department of Pediatrics, Division of Rheumatology, Hacettepe University Faculty of Medicine, Ankara, Turkey); D. Kipgen (MD,Pathology Department, Queen Elizabeth University Hospital, Glasgow, United Kingdom); D. Casartelli (Pathology Department, Manzoni Hospital, Lecco, Italy); D. Galesic Ljubanovic (MD, Nephrology Department, University Hospital, Zagreb, Croatia; Zagreb, Croatia); H Gakiopoulou (MD, Department of Pathology, National and Kapodistrian University of Athens, Athens, Greece); E. Bertoni (MD, Nephrology Department, Careggi Hospital, Florence, Italy); P. Cannata Ortiz (MD, Pathology Department, IIS-Fundacion Jimenez Diaz UAM, Madrid, Spain); H. KarkoszkaMD, (Nephrology, Endocrinology and Metabolic Diseases, Medical University of Silesia, Katowice, Katowice, Poland); H.J. Groene (MD, Cellular and Molecular Pathology, German Cancer Research Center, Heidelberg, Germany); A. Stoppacciaro (MD, Surgical Pathology Units, Department of Clinical and Molecular Medicine, Ospedale Sant'Andrea, Sapienza University of Rome, Rome, Italy); I. Bajema (MD, Department of Pathology, Leiden University Medical Center, Leiden, The Netherlands); J. Bruijn (MD, Department of Pathology, Leiden University Medical Center, Leiden, The Netherlands); X. Fulladosa Oliveras (MD, Nephrology Unit, Bellvitge University Hospital, Hospitalet de Llobregat, Barcelona, Spain); J. Maldyk (MD, Division of Pathomorphology, Children's Clinical Hospital, Medical University ofWarsaw,Warsaw, Poland); and E. Ioachim (MD, Department of Pathology, Medical School, University of Ioannina, Ioannina, Greece); the Oxford derivation and North American validation investigators: Bavbek N (MD, Department of Pathology, Vanderbilt University, Nashville, Tennessee); Cook T (MD, Imperial College, London, England), Troyanov S (MD, Division of Nephrology, Department of Medicine, Hopital du Sacre-Coeur de Montreal, Montreal, Quebec, Canada); Alpers C (MD, Department of Pathology, University of Washington Medical Center, Seattle,Washington), Amore A (MD, Nephrology, Dialysis and Transplantation Unit, Regina Margherita Children's Hospital, University of Turin, Turin, Italy), Barratt J (MD, The John Walls Renal Unit, Leicester General Hospital, Leicester, England); Berthoux F (MD, Department of Nephrology, Dialysis, and Renal Transplantation, Hôpital Nord, CHU de Saint-Etienne, Saint-Etienne, France); Bonsib S (MD, Department of Pathology, LSU Health Sciences Center, Shreveport, Los Angeles); Bruijn J (MD, Department of Pathology, Leiden University Medical Center, Leiden, The Netherlands); D'Agati V (MD, Department of Pathology, Columbia University College of Physicians Surgeons, New York, New York); D'Amico G (MD, Fondazione D'Amico per la Ricerca sulle Malattie Renali, Milan, Italy); Emancipator S (MD, Department of Pathology, Case Western Reserve University, Cleveland, Ohio); Emmal F (MD, Division of Nephrology and Dialysis, Department of Nephrology and Urology, Bambino Gesù Children's Hospital and Research Institute, Piazza S Onofrio, Rome, Italy); Ferrario F (MD, Renal Immunopathology Center, San Carlo Borromeo Hospital, Milan, Italy); Fervenza F (MD PhD, Division of Nephrology and Hypertension, Mayo Clinic, Rochester); Florquin S (MD, Department of Pathology, Academic Medical Center, University of Amsterdam, Amsterdam, The Netherlands); Fogo A (MD, Department of Pathology, Vanderbilt University, Nashville, Tennessee); Geddes C (MD, The Renal Unit, Western Infirmary, Glasgow, Scotland); Groene H (MD, Department of Cellular and Molecular Pathology, German Cancer Research Center, Heidelberg, Germany); Haas M(MD, Department of Pathology and Laboratory Medicine, Cedars-Sinai Medical Center, Los Angeles, California); Hill P (MD, St Vincent's Hospital, Melbourne, Australia); Hogg R (MD, Scott and White Medical Center, Temple, Texas (retired)); Hsu S (MD, Division of Nephrology, Hypertension and Renal Transplantation, College of Medicine, University of Florida, Gainesville, Florida); Hunley T (MD, Department of Pathology, Vanderbilt University, Nashville, Tennessee); Hladunewich (MD, Division of Nephrology, Sunnybrook Health Science Center, University of Toronto, Ontario, Canada M); Jennette C (MD, Department of Pathology and Laboratory Medicine, University of North Carolina, Chapel Hill, North Carolina); Joh K (MD, Division of Immunopathology, Clinical Research Center Chiba, East National Hospital, Chiba, Japan); Julian B (MD, Department of Medicine, University of Alabama at Birmingham, Birmingham, Alabama); Kawamura T (MD, Division of Nephrology and Hypertension, Jikei University School of Medicine, Tokyo, Japan); Lai F (MD, The Chinese University of Hong Kong, Hong Kong); Leung C (MD, Department of Medicine, Prince of Wales Hospital, Chinese University of Hong Kong, Hong Kong); Li L (MD, Research Institute of Nephrology, Jinling Hospital, Nanjing University School of Medicine, Nanjing, China); Li P (MD, Department of Medicine, Prince of Wales Hospital, Chinese University of Hong Kong, Hong Kong); Liu Z (MD, Research Institute of Nephrology, Jinling Hospital, Nanjing University School of Medicine, Nanjing, China); Massat A (MD, Division of Nephrology and Hypertension, Mayo Clinic, Rochester, Minnesota); Mackinnon B (MD, The Renal Unit,Western Infirmary, Glasgow, Scotland); Mezzano S (MD, Departamento de Nefrología, Escuela de Medicina, Universidad Austral, Valdivia, Chile); Schena F (MD, Renal, Dialysis and Transplant Unit, Policlinico, Bari, Italy); Tomino Y (MD, Division of Nephrology, Department of Internal Medicine, Juntendo University School of Medicine, Tokyo, Japan); Walker P (MD, Nephropathology Associates, Little Rock, Arkansas);Wang H (MD, Renal Division of Peking University First Hospital, Peking University Institute of Nephrology, Beijing, China (deceased)); Weening J (MD, Erasmus Medical Center, Rotterdam, The Netherlands); and Yoshikawa N (MD, Department of Pediatrics, Wakayama Medical University, Wakayama City, Japan); the International investigators: Cai-Hong Zeng (MD, Nanjing University School of Medicine, Nanjing, China); Sufang Shi (MD, Peking University Institute of Nephrology, Beijing, China); C.Nogi (MD, Juntendo University, Faculty of Medicine, Tokyo, Japan); H.Suzuki (MD, Juntendo University, Faculty of Medicine, Tokyo, Japan); K. Koike (MD, Division of Nephrology and Hypertension, Department of Internal Medicine, Jikei University School of Medicine, Tokyo, Japan); K. Hirano (MD, Division

of Nephrology and Hypertension, Department of Internal Medicine, Jikei University School of Medicine, Tokyo, Japan); T. Kawamura (MD, Division of Nephrology and Hypertension, Department of Internal Medicine, Jikei University School of Medicine, Tokyo, Japan); T. Yokoo (MD, Division of Nephrology and Hypertension, Department of Internal Medicine, Jikei UniversitySchool of Medicine, Tokyo, Japan); M. Hanai (MD, Division of Nephrology, Department of Medicine, Kurume University School of Medicine, Fukuoka, Japan); K. Fukami (MD, Division of Nephrology, Department of Medicine, Kurume University School of Medicine, Fukuoka,, Japan); K. Takahashi (MD, Department of Nephrology, Fujita Health University School of Medicne, Aichi, Japan); Y. Yuzawa (MD, Department of Nephrology, Fujita Health University School of Medicine, Aichi, Japan); M. Niwa (MD, Department of Nephrology, Nagoya University Graduate School of Medicine, Aichi, Japan); Y. Yasuda (MD, Department of Nephrology, Nagoya University Graduate School of Medicine, Aichi, Japan); S. Maruyama (MD, Department of Nephrology, Nagoya University Graduate School of Medicine, Aichi, Japan); D. Ichikawa (MD, Division of Nephrology and Hypertension, Department of Internal Medicine, St Marianna University School of Medicine, Kanagawa, Japan); T. Suzuki (MD, Division of Nephrology and Hypertension, Department of Internal Medicine, St Marianna University School of Medicine, Kanagawa, Japan); S. Shirai (MD, Division of Nephrology and Hypertension, Department of Internal Medicine, St Marianna University School of Medicine, Kanagawa, Japan); A. Fukuda (MD, First Department of Internal Medicine, Faculty of Medicine, University of Miyazaki, Miyazaki, Japan); S. Fujimoto (MD, Department of Hemovascular Medicine and Artificial Organs, Faculty of Medicine, University of Miyazaki, Miyazaki, Japan); H. Trimarchi (MD, Division of Nephrology, Hospital Britanico, Buenos Aires, Argentina).
