# Supplementary material for: Detection of PatIent-Level distances from single cell genomics and pathomics data with Optimal Transport (PILOT)
Source: Mol Syst Biol. 2023 Dec 19;20(2):57–74. doi: 10.1038/s44320-023-00003-8 (PMC10883279; doi:10.1038/s44320-023-00003-8)
Supplement: Supplementary file 7 — Expanded View Figures [file 44320_2023_3_MOESM7_ESM.pdf]

Expanded View Figures

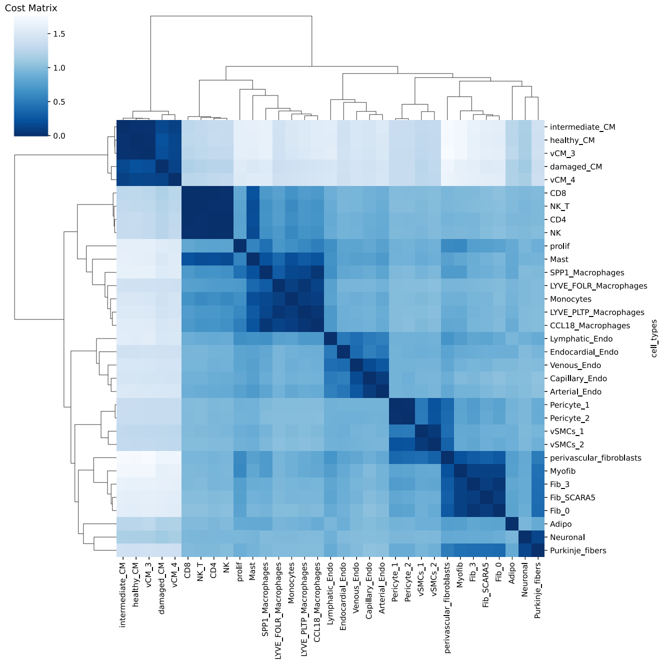

Figure EV1. Example of cost matrix used for optimal transport in the myocardial infarction scRNA-seq data.

The cost matrix obtained at the analysis of the myocardial infarction based on 33 cell clusters. The cost (or distance) between cardiomyocyte cell types is lower to each other (healthy-CM, intermediary-CM, damaged-CM) than when compared with fibroblast cells.

© The Author(s)

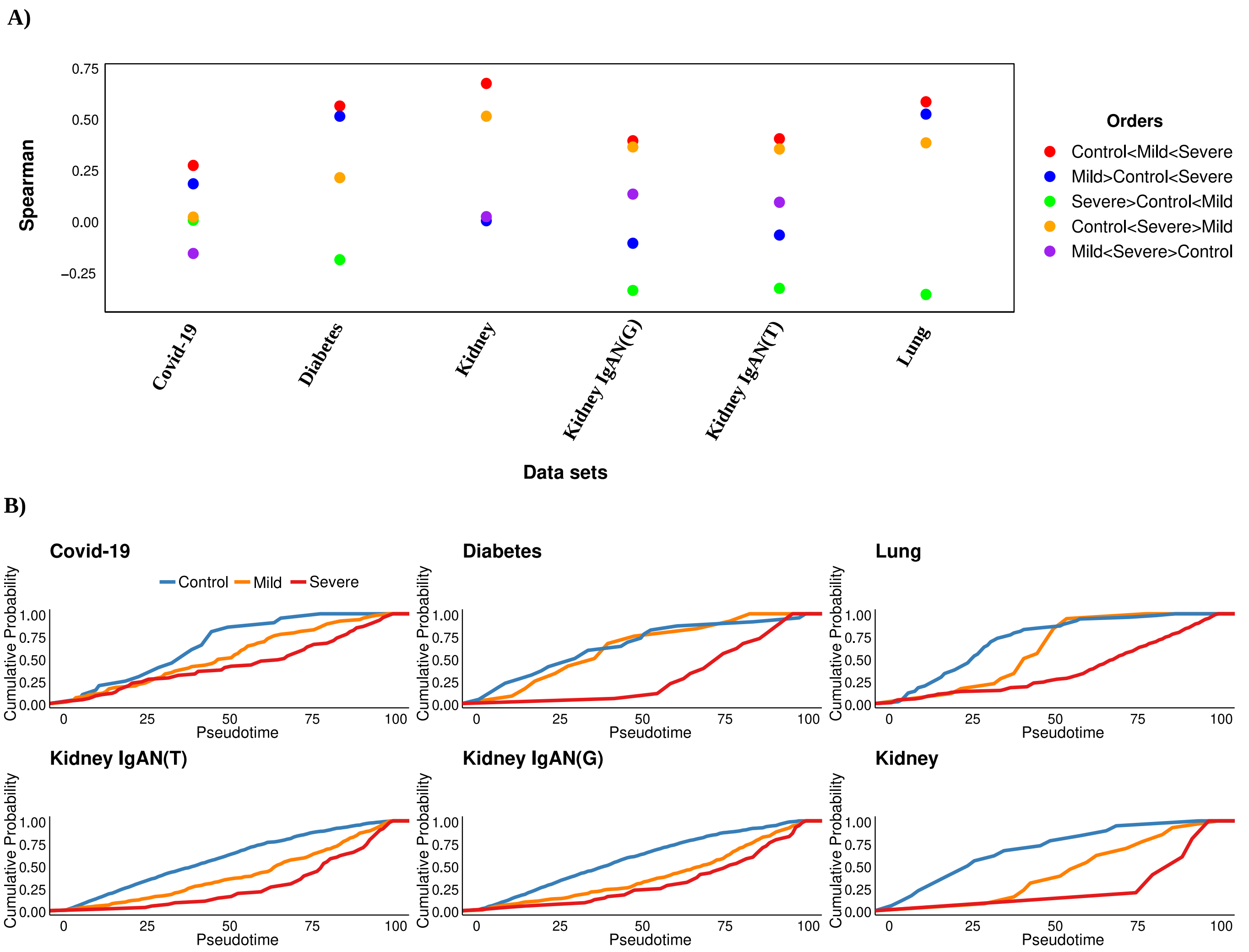

Figure EV2. Benchmarking analysis of trajectories and disease progression stage.

(**A**) Spearman Correlation (y-axis) between disease progression scores and ordered classes for distinct data sets (x-axis) by using PILOT. We systematically shuffled the order of control, mild and severe samples to investigate if the order is capture by the algorithms. We observe highest Spearman correlation values for the order "control < mild < severe" in all data sets. (**B**) Cumulative probability of control, mild and severe cases (y-axis) over PILOT estimated pseudotime (x-axis) for all multi-class data sets.

 © The Author(s)

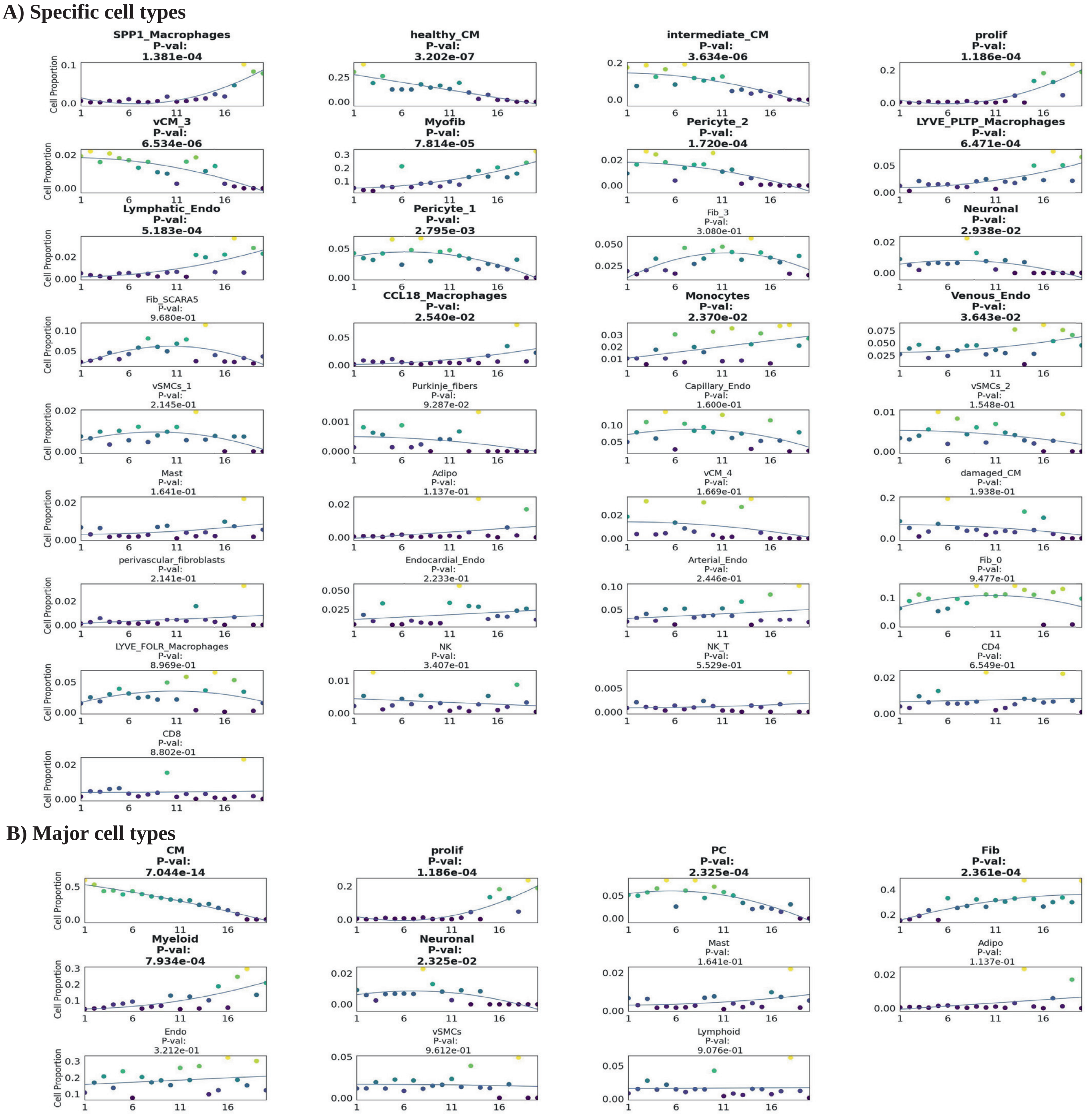

Figure EV3. Cell composition changes in the myocardial infarction scRNA-seq data.

Cell cluster frequency (y-axis) vs. PILOT disease progression (x-axis) for Myocardial Infarction scRNA-seq data for high granularity clusters ($k = 33$) (**A**) and low granularity clusters ($k = 11$) (**B**). Cells with a significant association with pseudotime are marked in bold. *p*-value of (**A**, **B**) were estimated with the F-statistic test.

© The Author(s)

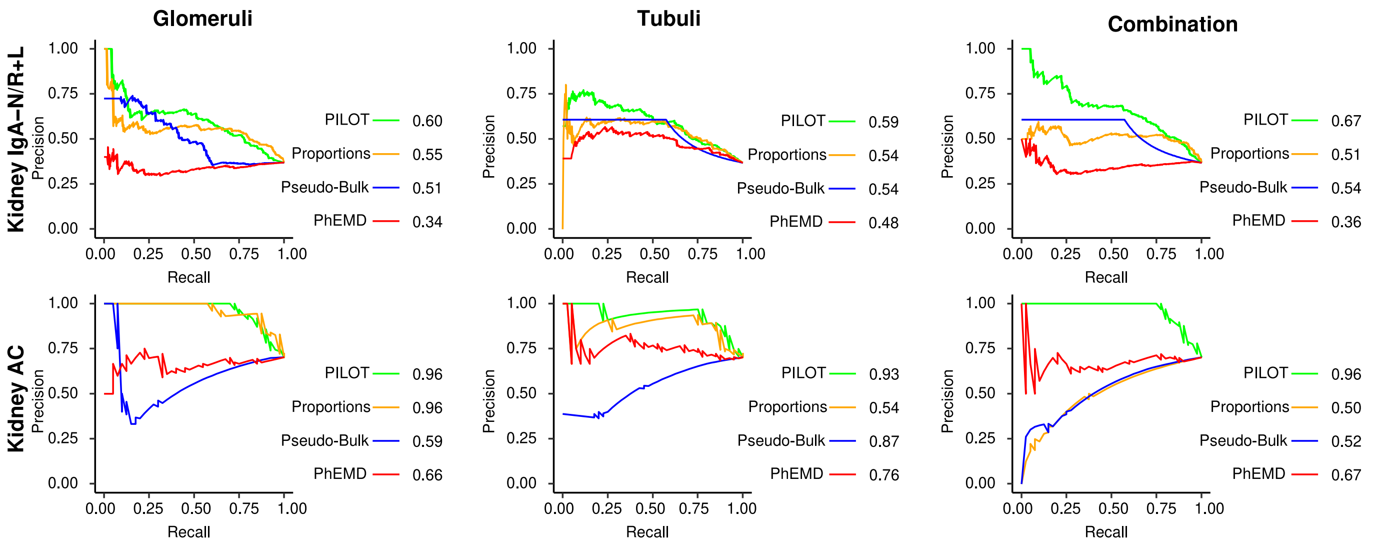

Figure EV4. Trajectory prediction scores for Kidney pathomics data sets.

AUCPR plots for Glomeruli, Tubule and both (combined) for Kidney AC, Kidney IgAN pathomics data.

 © The Author(s)
